# Enhancing Functional Recovery After Spinal Cord Injury Through Neuroplasticity: A Comprehensive Review

**DOI:** 10.3390/ijms26146596

**Published:** 2025-07-09

**Authors:** Yuan-Yuan Wu, Yi-Meng Gao, Ting Feng, Jia-Sheng Rao, Can Zhao

**Affiliations:** 1 Beijing Key Laboratory for Biomaterials and Neural Regeneration, National Medical Innovation Platform for Industry-Education Integration in Advanced Medical Devices (Interdiscipline of Medicine and Engineering), School of Biological Science and Medical Engineering, Beihang University, Beijing 100191, China; yuanywu@buaa.edu.cn (Y.-Y.W.); gym_summer@buaa.edu.cn (Y.-M.G.); fengt@buaa.edu.cn (T.F.); 2 Beijing International Cooperation Bases for Science and Technology on Biomaterials and Neural Regeneration, Beijing Advanced Innovation Center for Biomedical Engineering, Beihang University, Beijing 100191, China; 3 Institute of Rehabilitation Engineering, China Rehabilitation Science Institute, Beijing 100068, China

**Keywords:** spinal cord injury, neuroplasticity, functional recovery, treatment strategy

## Abstract

Spinal cord injury (SCI) is a severe neurological condition that typically results in irreversible loss of motor and sensory function. Emerging evidence indicates that neuroplasticity, the ability of the nervous system to reorganize by forming new neural connections, plays a pivotal role in structural and functional recovery post-injury. This insight lays the groundwork for the development of rehabilitation and therapeutic strategies designed to leverage neuroplasticity. In this review, we offer an exhaustive overview of the neuroplastic alterations and mechanisms that occur following an SCI. We examine the role of neuroplasticity in functional recovery and outline therapeutic approaches designed to augment neuroplasticity post-SCI. The process of neuroplasticity post-SCI involves several physiological processes, such as neurogenesis, synaptic remodeling, dendritic spine formation, and axonal sprouting. Together, these processes contribute to the reestablishment of neural circuits and functional restoration. Enhancing neuroplasticity is a promising strategy for improving functional outcomes post-SCI; however, its effectiveness is influenced by numerous factors, including age, injury severity, time since the injury, and the specific therapeutic interventions employed. A variety of strategies have been suggested to promote neuroplasticity and expedite recovery, including pharmacological treatments, biomaterial-based therapies, gene editing, stem cell transplantation, and rehabilitative training. The combination of personalized rehabilitation programs with innovative therapeutic techniques holds considerable potential for maximizing the benefits of neuroplasticity and enhancing clinical outcomes in SCI management.

## 1. Introduction

Spinal cord injury (SCI), typically resulting from external trauma or disease, is one of the most devastating injuries of the central nervous system (CNS). It often leads to the loss of sensory, motor, and autonomic functions [1]. In 2019, it was estimated that globally there were 900,000 new cases of SCI, cumulatively involving 20.6 million individuals living with the condition and 6.2 million experiencing resultant disabilities [2]. The necessity for long-term medical care and rehabilitation for patients with spinal cord injury creates a substantial economic and healthcare burden worldwide. In Canada alone, the annual economic cost of traumatic SCI is estimated at USD2.67 billion, with lifetime expenses ranging from USD1.5 million for individuals with incomplete paraplegia to USD 3 million for those with complete quadriplegia [3,4]. Consequently, the scientific prevention, treatment, and rehabilitation of SCI, as well as the mitigation of their adverse economic and social impacts, remain key research areas within the field of spinal cord rehabilitation.

When injury to the spinal cord occurs, it not only disrupts the flow of information between the brain and regions below the injury site but also instigates a complex series of structural and functional alterations that impact the balance of the entire nervous system. During the acute phase of SCI, mechanical damage and subsequent inflammatory responses contribute to axonal disruption, degeneration, demyelination, and neuronal cell death within neural circuits [5,6]. As the injury progresses into the chronic phase, the formation of glial scars and the enlargement of cysts further hinder nerve regeneration [7,8,9]. However, the CNS maintains a certain degree of plasticity, which provides the potential for functional compensation post-injury [10,11]. This compensation allows patients to regain functional independence by adapting to the loss of voluntary muscle control below the lesion site and employing alternative strategies and assistive technologies. This is particularly significant in severe SCI cases, where voluntary motor function is absent below the level of injury [12,13]. After injury, residual neurons can partially recover their function via synaptic remodeling and neural network reorganization. This process involves the secretion of neurotrophic factors, cytokine regulation, and morphological alterations in nerve cells. Together, these promote the formation of new synapses and neural circuits, aiding in functional compensation and recovery [14]. It is noteworthy that functional recovery following SCI hinges not only on local repair but also on the reorganization and compensation of the cerebral cortex. These synergistic mechanisms collaboratively contribute to the restoration of neurological functions post-injury [15]. The intrinsic regenerative ability of the spinal cord post-injury is admittedly limited. However, therapeutic interventions promoting neural repair and regeneration could potentially restore some measure of spinal cord functionality. Hence, a comprehensive understanding of the structural and functional changes after SCI is paramount for developing effective treatment strategies and enhancing functional recovery.

Moreover, an individual’s neuroplasticity—the ability for adaptive reorganization of neural circuits and synaptic connections—largely influences recovery and functional improvement after a SCI. Existing evidence increasingly suggests that the CNS exhibits remarkable neuroplastic potential, thereby providing a promising foundation for functional recovery post-injury [10]. While therapeutic approaches designed to stimulate neural regeneration following SCI are still in the preclinical or early clinical stages, leveraging the neuroplasticity of the CNS presents significant potential for enhancing functional restoration. This review will encapsulate the mechanisms of neuroplasticity and their role in SCI recovery, and will further explore how various therapeutic strategies could impact neuroplastic responses.

## 2. Mechanisms of Neuroplasticity

### 2.1. Definition of Neuroplasticity

Neuroplasticity involves adaptive structural and functional modifications in the CNS, enabling the system to reorganize its activity in response to intrinsic or extrinsic stimuli [16]. This process drives significant nervous system changes and is pivotal for development, lifelong learning [17], as well as memory formation and recovery from injury or disease [18]. However, neuroplasticity can also have adverse effects. For example, after SCI, the emergence and/or enhancement of aberrant neural connections can contribute to or exacerbate phantom pain, thus impeding the patient’s functional recovery and accelerating disease progression [19].

Neuroplasticity following SCI includes both functional and structural aspects. Typically, functional reorganization at the mesoscopic level is supported by structural changes that are governed by various physiological events occurring at the microscale, such as neurogenesis, synaptic remodeling, dendritic spine formation, and axonal sprouting. Functional and structural plasticity are often interdependent—molecular and cellular changes support systems-level reorganization [20,21,22].

#### 2.1.1. Functional Plasticity

Functional plasticity refers to changes in neural communication and processing. A wealth of evidence indicates that functional plasticity following SCI takes place at multiple levels, such as the local injured cord, peripheral nervous system (PNS), and supraspinal centers (including brainstem and cerebral structures) [11,23]. The functional reorganization of these areas compensates for denervated/deafferented regions through processes like cortical remapping of brain functions to alternative areas or cross-modal redistribution of homologous regions. This allows the nervous system to re-establish the “brain–spinal cord–target organ” projection pathways, aiding in functional recovery. Most research in this field has concentrated on functional plasticity at the brain level after SCI, with fewer studies looking into plasticity mechanisms at other levels or the interaction between different levels. Nevertheless, some studies have shown that spinal-level plasticity can enhance supraspinal functional remodeling to restore neurological functions after injury [24].

#### 2.1.2. Structural Plasticity

Structural plasticity refers to the physical changes that occur in neural circuits, such as axonal sprouting and synaptic remodeling [25]. Following SCI, intramedullary structural changes to neuronal somata, axons, dendrites, and synaptic architectures may reinforce existing neural pathways or help to establish new neural circuits to improve functional recovery [11]. Previous experimental studies have shown that focal activation of certain spinal interneurons can trigger structural reorganization of the spinal cord independently of injury status, even in the normal spinal cord [26]. This suggests that neuroplasticity is an inherent feature of the nervous system that can occur regardless of the current state of the tissue. After SCI, structural plasticity may also occur at supraspinal levels, including the brain stem and cortical regions, comprising changes to neural connectivity and brain regions that are involved in functional recovery [24].

##### Neuronal Changes After SCI

Neurons, which are the fundamental structural and functional units of the nervous system, consist of three main components: the soma, dendrites, and axon. These cells are specifically adapted to receive, transmit, and process neural information. They coordinate complex physiological functions such as sensory perception, motor control, cognition, and memory formation through intricate network connectivity [27].

After SCI, neuronal populations experience significant quantitative changes due to primary and secondary injury mechanisms, with neuronal loss and apoptosis being the predominant contributors [28,29,30]. The initial mechanical trauma directly affects neurons and axons at the lesion site, leading to acute neuronal depletion within the central injury area. Over time, secondary injury mechanisms such as ischemia–hypoxia, excitotoxicity, and oxidative stress exacerbate this process. These mechanisms induce mitochondrial dysfunction and activate apoptotic pathways, triggering programmed cell death in surviving peri-lesional neurons and further reducing neuronal density. Concurrently, axonal disconnection initiates Wallerian degeneration, a process characterized by the progressive disintegration of the axon-myelin complex distal to the primary injury site. This trans-synaptic degeneration not only causes retrograde damage to upstream neurons but also promotes secondary neuronal loss through the deprivation of neurotrophic support to distal neurons [31,32]. The disruption of brain–spinal cord descending tracts reduces synaptic input to motor neurons below the injury level, driving postsynaptic structural remodeling. This remodeling is marked by dendritic spine elimination and synaptic deprivation-induced atrophy, key pathological substrates of motor dysfunction and impaired functional recovery [6,33,34,35]. Furthermore, reactive glial proliferation replaces the space occupied by dead neurons, further altering local tissue structure and adversely affecting neuronal function [36,37]. Ultimately, metabolic crises such as dysregulation of energy metabolism and calcium overload induce ultrastructural neuronal damage and impair the capacity for self-repair. This creates a negative feedback loop that collectively drives irreversible reductions in neuronal density and the collapse of functional networks [38,39]. Experimental evidence reveals that neuronal atrophy in the pelvic ganglion can be observed as early as one week after SCI [40]. Additionally, another study has found that SCI can cause temporary morphological changes in sympathetic preganglionic neurons, including loss of dendrites and reduction in neuronal volume below the injury site. These changes may be partially reversed over time [41].

In the injured area, neurons frequently exhibit hallmark degenerative changes, including nuclear condensation and cytoplasmic vacuolization. Nuclear condensation, commonly linked to ischemia, inflammation, or apoptosis and serves as a significant indicator of neuronal degeneration [42,43]. Cytoplasmic vacuolization typically results from cellular stress pathways, such as endoplasmic reticulum stress and mitochondrial dysfunction. These processes disrupt cellular homeostasis, leading to structural damage and functional loss of neurons [44]. Furthermore, axonal and dendritic degeneration play pivotal roles in the secondary injury mechanism. Axonal disintegration manifests as fragmentation and shortening, interfering with interneuronal communication and axonal transport systems, ultimately resulting in a substantial decrease in neurological function [45,46]. Dendritic degeneration reduces synaptic inputs, impairs neuronal signal processing capability, and further disrupts synaptic transmission and network connectivity [47]. Despite these pathological changes, the injured cord retains an inherent adaptive plasticity, initiating synaptic reorganization and dendritic spine remodeling to enhance synaptic transmission and restore some functions [47,48].

##### Axonal Changes After SCI

The axons of neurons are responsible for transmitting efferent signals and typically terminate at axonal endings [49]. After SCI, the pathological alterations observed in axons and their associated plasticity mechanisms become central topics in the field of neural repair and functional recovery.

After SCI, axons undergo substantial structural and functional changes that are intricately regulated by neural plasticity. The early pathological alterations primarily encompass Wallerian degeneration in the distal axonal segment and retrograde degeneration proximally. Both these changes are marked by a cytoskeletal collapse and disruption of axonal transport, leading to neuronal dysfunction or even cell death [50,51,52,53]. Although the regenerative ability of the adult CNS is intrinsically limited, the activation of regeneration-associated signaling pathways, such as growth-associated protein 43 (GAP-43) and activating transcription factor 3 (ATF3), can stimulate limited axonal regrowth early after the injury [54,55]. Moreover, some spared neurons can experience axonal collateral sprouting to form new synaptic connections, contributing to circuit reorganization and partial functional compensation [56,57]. These alterations highlight the pivotal role of neural plasticity in axonal remodeling. At the synaptic level, plasticity mechanisms like long-term potentiation (LTP) and long-term depression enhance or inhibit synaptic transmission efficiency, playing a crucial role in functional recovery [58]. On the molecular level, neurotrophic factors such as brain-derived neurotrophic factor (BDNF) and neurotrophin-3 (NT-3) promote axonal extension by activating the MAPK/ERK and PI3K/Akt pathways downstream of Trk receptors [59,60,61,62]. Additionally, extracellular matrix components like laminin and fibronectin provide essential structural support for axonal growth [63,64,65,66]. Notably, the elimination of inhibitory signaling pathways, particularly the Nogo-A/NgR [67,68] and Rho/ROCK [69,70] pathways, has emerged as a key strategy for promoting axonal regeneration.

##### Changes in Dendritic Spines After SCI

Dendrites primarily receive signals from other neurons and are crucial for local protein synthesis and independent signal processing, making them vital structures for neuronal information integration [71]. Dendritic spines, micron-scale protrusions on dendritic shafts, modulate synaptic transmission efficiency. After SCI, there is a notable decrease in dendritic spine density, especially near the lesion site. Yu et al. [47,72] showed in a *macaque* model of SCI that corticospinal tract (CST) neurons in motor-related regions exhibit diminished basal dendrite complexity and a 20–40% reduction in dendritic spine density. These changes were particularly significant in the primary motor cortex and supplementary motor area, potentially hindering motor function recovery. It is worth noting that dendritic spines can undergo remodeling post-SCI. Research indicates that under specific conditions, the quantity of dendritic spines can increase, especially the mature mushroom-shaped ones [70]. For example, after peripheral nerve injury, slender dendritic spines on lamina II neurons in the dorsal horn of the spinal cord transform into mature mushroom-type spines, and the density of these spines rises over time. This correlates with improved synaptic efficiency and fidelity but may also lead to pathological hyperexcitability associated with chronic pain [47,73,74].

##### Synaptic Changes After SCI

Synapses, the fundamental structures facilitating information transmission between neurons or between neurons and their target cells, also play a pivotal role in maintaining the functional stability of neural networks and enabling neural plasticity. SCI markedly impacts the structure and function of both central and peripheral neural circuits. Within the CNS, an SCI triggers abnormalities in excitability and synaptic transmission in local spinal circuits. Conversely, in the PNS, it often leads to dysfunction of autonomic neurons innervating target organs in the affected region [33,75]. Notably, structural and functional alterations at the synaptic level represent a significant component of the secondary injury cascade, playing a crucial role in neural plasticity. These modifications encompass synaptic loss, structural remodeling, and compensatory reorganization, engaging both excitatory and inhibitory synaptic inputs, thereby influencing neural circuit functionality [76]. Furthermore, an SCI modifies the functional and physiological attributes of synapses, particularly in autonomic neurons. For example, in the acute phase of injury, excitatory postsynaptic potentials (EPSPs) in autonomic neurons exhibit extended rise times and decay time constants. Conversely, in the chronic phase, EPSP amplitudes diminish while rise and decay times are abbreviated, correlating with altered expression of postsynaptic nicotinic receptor subunits, ultimately impacting neural signal transmission and function [75]. Although extensive synaptic loss transpires in the early phases of an SCI, the gradual establishment of new synaptic connections may facilitate functional recovery over time [77]. Nevertheless, this remodeling process might also precipitate maladaptive plasticity. For example, heightened excitability of nociceptive pathways following an SCI is intimately linked with the onset of chronic pain conditions such as paresthesia pain syndromes. Therefore, a comprehensive understanding of the synaptic structural and functional modifications following SCI is imperative. Such insights will facilitate the identification of refined therapeutic targets and augment the precision and efficacy of rehabilitation interventions.

### 2.2. Mechanisms of Neural Plasticity

Compared to the PNS, the CNS displays a restricted ability for spontaneous regeneration following SCI, largely due to inhibitory molecules such as chondroitin sulfate proteoglycans (CSPGs) and myelin-associated inhibitors [6,78]. However, some neural plasticity is evident after SCI, with the primary mechanisms being axonal regeneration, neuronal sprouting, and circuit reorganization. These processes are influenced by cellular mechanisms regulated by molecular signals, encompassing dynamic modulation of growth factors, signaling pathways, and cytoskeletal organization.

#### 2.2.1. Remodeling Mechanisms at the Cellular Level

After SCI, a rapid activation of various glial cells within the CNS occurs across distinct temporal scales. These cells collectively contribute to tissue repair and functional reorganization. The activation and degeneration of glial cells are critical elements in the patho-physiological changes that occur acutely following an SCI [79]. Furthermore, their immunomodulatory interactions may play a key role in promoting neural regeneration and repair [80]. Studies have shown that activated astrocytes regulate the proliferation, differentiation, and maturation of oligodendrocyte precursor cells (OPCs), thus impacting myelin regeneration. Simultaneously, activated microglia modulate post-SCI inflammatory responses, further influencing remyelination processes [81]. Moreover, glial cells secrete pro-sprouting molecules (e.g., osteopontin and plasminogen activator), which are upregulated in denervated regions to mimic developmental cues [82]. However, some responses, such as glial scar formation and the release of inflammatory cytokines, may partially inhibit axonal regeneration [9]. Recent studies have also shown that the transplantation of neural progenitor cells can reprogram adult neurons into an embryonic-like state, facilitating CST axonal regeneration and synaptic reformation. This process involves the activation of multiple transcription factors (e.g., ATF3, SOX11, and STAT3), which reactivate growth-associated gene expression programs, enhance neuronal plasticity, and promote neural circuit reconstruction [83].

##### Astrocytes

Astrocytes, a prominent glial cell type in the CNS, are ubiquitously present in the brain and spinal cord, where they perform multifaceted roles such as supporting, protecting, and regulating neuronal function [84]. After SCI, astrocytes are swiftly activated and contribute to tissue repair, inflammation regulation, and neural regeneration. Research suggests that Stat3 acts as a key transcription factor that regulates the functions of reactive astrocytes post-SCI, thus representing a potential therapeutic target for treating CNS injuries [85]. Astrocytes have the ability to produce and secrete various proteins and neurotrophic factors. For instance, proteins like thrombospondins can bind to the α2δ-1 receptor on neurons, promoting synapse formation and maturation [86,87,88]. Similarly, the protein agrin has also been demonstrated to facilitate synaptogenesis and neuronal branching [89,90]. Neurotrophic factors secreted by astrocytes include BDNF, NT-3, and ciliary neurotrophic factor (CNTF), among others. Notably, BDNF promotes neuronal branching and synapse formation by activating the tropomyosin receptor kinase B (TrkB) receptor and plays a crucial role in maintaining LTP, thereby contributing to learning and memory [91]. NT-3 activates the TrkC receptor and supports neuronal survival and growth by enhancing dendritic regeneration and synaptic plasticity [92]. CNTF has been shown to promote neuronal survival and axon regeneration [93]. Furthermore, astrocytes contribute to extracellular homeostasis by clearing neurotoxic proteins and metabolites [94]. After SCI, reactive astrocytes generate dense glial scars surrounding the lesion core [36,37]. During the acute phase, these scars curtail the spread of inflammation, safeguard the neurons and tissues that have been spared, and provide structural support for neural regeneration [95,96]. Despite their role in stabilizing secondary injury progression, glial scars can also serve as physical and chemical impediments to axonal regrowth [97,98]. Lin et al. [99] illustrated that inhibitors of MEK may suppress astrocyte proliferation, reduce glial scar formation, and enhance hindlimb motor function. Faulkner et al. [100] reported in transgenic *mouse* models that eliminating reactive astrocytes post-injury results in blood–spinal cord barrier disruption, severe demyelination, neuronal and oligodendrocyte death, and significant motor deficits. This underscores the critical protective roles that reactive astrocytes play in tissue preservation and functional maintenance. Activation of nuclear factor erythroid 2-related factor 2 in astrocytes, the primary transcriptional regulator of cellular antioxidant responses, alleviates oxidative stress and neuroinflammation while bestowing neuroprotective effects post-SCI [101]. Therefore, astrocytes exhibit a dual role post-SCI, promoting neural repair but also limiting regeneration.

##### Oligodendrocytes

Oligodendrocytes (OLs) are essential glial cells in the CNS, mainly originating from OPCs situated in the embryonic spinal cord and the ventral ventricular zone of the forebrain [95,96]. Collectively, OLs and OPCs play a significant role in myelin formation and provide substantial support to axons in the healthy CNS. Following an SCI, there is substantial cell death among OLs, leading to extensive demyelination, which adversely affects the function and survival of axons. Subsequently, OPCs are swiftly activated and proliferate with the intent to remyelinate the damaged axons and thus facilitate neurological recovery [37].

OPCs are the most prevalent and rapidly proliferating progenitor cells in the adult CNS [102], serving as the primary source of remyelination post-SCI. Upon injury, OPCs are swiftly activated and proliferate, predominantly at the lesion site and its periphery. Subsequently, they differentiate into mature oligodendrocytes, contributing to the remyelination process [103]. The proliferation of endogenous OPCs may be a compensatory response to the loss of damaged OLs. Moreover, transplantation of exogenous OPCs has been observed to mitigate SCI-induced neuropathic pain and promote remyelination [104]. However, endogenous remyelination post-trauma is frequently incomplete [105]. Studies have indicated that after SCI, OPCs are not only significantly activated at the injury site but also in distant regions (e.g., lumbar bulge), evidenced by elevated expression of OPC markers (NG2), oligodendroglial lineage markers, and myelin proteins [106]. OPCs aid in endogenous regeneration and display therapeutic promise when exogenously transplanted. Studies reveal that the transplantation of human embryonic stem cell-derived OPCs shortly after injury (i.e., 7 days) results in significant cell survival, widespread migration, and maturation into oligodendrocytes. This process enhances remyelination and improves motor function. However, transplantation at later stages (e.g., 10 months) significantly diminishes therapeutic efficacy [107]. It is worth noting that while OPCs promote remyelination, they also contribute to glial scar formation, which can impede axonal regeneration [102,105]. Zhao et al. [106] demonstrated that post-SCI OPCs exhibit neurogenic potential and show considerable activation of endoplasmic reticulum (ER) stress. Inhibition of ER stress effectively reduces OPCs’ mortality. Their findings suggest that the ER may regulate the stemness and differentiation of oligospheres. Apart from myelination, OLs transfer energy metabolites such as lactate and pyruvate to axons via monocarboxylate transporter 1 [108,109]. Also, NG2 glia have been shown to promote axonal regeneration within and beyond the lesion site [110], suggesting their wider roles in SCI repair. Notably, Lee et al. [111] employed a direct reprogramming strategy to convert fibroblasts into directly induced neural stem cells, which were further differentiated into proliferative OPCs (DN-OPCs). Transplantation of DN-OPCs in animal models has been shown to significantly enhance motor functional recovery and remyelination in *rats* with SCI, underscoring the therapeutic potential of OPCs in neural repair.

##### Microglia

Microglia, the intrinsic immune cells of the CNS, are swiftly activated following CNS injury. They play pivotal roles in maintaining tissue homeostasis by eliminating pathogens and cellular debris, and by secreting pro-repair cytokines and neurotrophic factors [112,113,114]. Moreover, microglia contribute to neurogenesis, axonal growth, and remyelination, thereby aiding neural network remodeling and functional recovery [115,116]. However, there is a potential for microglia to display excessive activation and cytotoxicity during the repair process, which may elevate the risk of secondary damage [117,118]. In *rodent* models, microglia are the first glial cells to respond to SCI, becoming activated within hours through proliferation and migration toward the lesion site [36,37]. Once activated, microglia can polarize into either pro-inflammatory (M1) or anti-inflammatory (M2) phenotypes. M1 microglia release pro-inflammatory cytokines such as IL-1β and TNF-α, potentially worsening inflammation and tissue damage, whereas M2 microglia secrete anti-inflammatory mediators like IL-10, promoting neuroprotection and tissue repair [119]. Additionally, microglia orchestrate neuroinflammatory responses by coordinating astrocytic activation and recruiting monocyte-derived macrophages to the injury site [115,119]. Transforming growth factor-β1 (TGF-β1), a key regulator of microglial homeostasis and CNS inflammation, is both produced by and acts upon microglia [120]. Notably, recent studies have shown that microglia can also prevent axonal degeneration by forming “protective caps” at the nodes of Ranvier, directly contacting and insulating axons to prevent fragmentation during the early phases of SCI [52].

#### 2.2.2. Molecular Regulatory Mechanisms

After SCI, the CNS instigates an intricate series of molecular regulatory processes. These processes involve a variety of signaling molecules and pathways that collaboratively modulate neural plasticity, forming the molecular foundation for injury repair. Among these processes, neurotrophic factors are pivotal for neural regeneration as they promote neuronal survival, axonal growth, and synapse formation. They primarily enhance neural plasticity through the activation of multiple intracellular signaling pathways, such as PI3K/AKT and MAPK cascades [121,122]. Additionally, transcription factors play a crucial role in regulating gene expression related to axonal regeneration, cytoskeletal remodeling, and axonal elongation post-SCI [123]. Furthermore, axon guidance molecules serve a bidirectional regulatory function in either guiding or restricting axon extension after injury, directing axons towards their target, or inhibiting their growth [124,125]. These molecular mechanisms work in tandem to influence the complex alterations in neural function following injury.

##### Neurotrophic Factor Signaling Pathways

Neurotrophic factors, pivotal regulatory molecules, orchestrate neuronal plasticity across developmental stages and adulthood, performing vital roles in neuronal survival, differentiation, axonal growth, and synaptic remodeling. Noteworthy neurotrophic factors encompass BDNF, NT-3, nerve growth factor (NGF), and glial cell line-derived neurotrophic factor (GDNF). These factors not only facilitate axonal elongation and branching but also promote neurite outgrowth and enhance synaptogenesis, thereby contributing significantly to the reconstruction of neural circuits. The process entails the reorganization of existing neuronal connections, as well as the formation of new ones, both of which are essential for replacing damaged pathways and restoring neurological function following SCI [61,122,126].

BDNF is widely recognized as a pivotal neurotrophic factor in neural repair after SCI, given its role in the regeneration of various neuronal populations, encompassing both brain and spinal cord axons [61,122]. When BDNF binds to its high-affinity receptor, TrkB, it triggers autophosphorylation of tyrosine residues, subsequently activating key intracellular signaling cascades such as MAPK/ERK, PI3K/Akt, and PLCγ. The MAPK/ERK pathway oversees cell proliferation, differentiation, and survival, while bolstering axonal regeneration and synaptic plasticity [122,127,128]. The PI3K/Akt and PLCγ pathways are associated with neuroprotection and plasticity. Specifically, the PI3K/Akt pathway inhibits neuronal apoptosis, sustains cellular metabolism, and facilitates axon elongation. In contrast, the PLCγ pathway amplifies neuronal excitability and synaptic plasticity via intracellular Ca^2+^ elevation and CaMKII activation [129]. Together, these interconnected mechanisms foster neuronal survival, axon growth, and synaptic remodeling. Additionally, BDNF concentration gradients can steer the growth of regenerating axons towards denervated targets [83], and exogenous BDNF supplementation has been demonstrated to markedly enhance neuronal regeneration and functional recovery [130].

NT-3 functions by activating tropomyosin receptor kinase C (TrkC) receptors, promoting axonal growth and neuronal differentiation, and facilitating the formation of new axonal connections, thereby aiding neural circuit reconstruction [131]. In a study utilizing a *monkey* SCI model, Rao et al. [132] demonstrated that NT-3, when combined with chitosan, enabled the long-distance regeneration of CSTs, leading to the restoration of both sensory and motor functions. Furthermore, NT-3 also modulates the excitability of motor neurons, providing additional support for neural circuit reorganization [126].

NGF plays a critical role in the interaction between the nervous and immune systems. It not only governs neuronal function but also influences the activity of immune cells. In numerous pathological states, there is a pronounced upregulation of NGF in inflamed tissues, which enhances peripheral innervation and neuronal excitability [133]. Such upregulation culminates in the release of neurotransmitters and immune-related neuropeptides, subsequently impacting both innate and adaptive immune responses. Beyond its immunomodulatory roles, NGF is indispensable for sensory nerve regeneration. Through activation of its high-affinity receptor, tropomyosin receptor kinase A, NGF ensures neuronal survival, encourages axonal growth, and stimulates the sprouting and regeneration of cholinergic motor neurons and nociceptive sensory fibers [61,134]. These attributes underscore NGF’s significance as a central signaling molecule in neuro–immune interactions and as a potential therapeutic target for inflammation-associated neural damage.

GDNF predominantly targets motor neurons, promoting their survival and axonal growth. Existing studies have demonstrated that the expression of GDNF by astrocytes is upregulated post-spinal cord ischemia, providing a protective mechanism for motor neurons against excitotoxic death [133]. Its primary mechanisms of action involve binding to the RET receptor and activating signaling cascades, such as the MAPK pathway, to promote neuronal survival and regeneration [135].

##### Regulation of Transcription Factors

After SCI, transcription factors are pivotal in regulating the gene expression linked to neuronal regeneration. The transcriptional response prompted by SCI encompasses various mechanisms and contributes to distinct phases of inflammation, repair, and regeneration [136].

During the acute phase, a series of transcription factors are rapidly activated. ATF3 is one of the earliest transcription factors upregulated after SCI. It is highly expressed in dorsal root ganglion neurons and can maintain the injured state for a prolonged period. It has also been reported that ATF3 could drive the injured state and promote axon regeneration and neurite growth [137,138]. Meanwhile, heme oxygenase 1, which exhibits anti-inflammatory and neuroprotective effects, showed the highest expression level on the third day after injury and reduced secondary damage by regulating the innate immune response of microglia [139]. In addition, many transcription factors were actively involved in regeneration-related pathways. In *zebrafish* models of SCI, Sox11b could induce neural differentiation of endogenous stem cells by activating neural stem cell-related genes such as *Nestin* and *Ascl1a* to promote spinal cord tissue regeneration [140]. Lymphoid enhancer-binding factor 1 (LEF1), a direct downstream effector of the Wnt/β-catenin pathway, mainly regulates cholesterol metabolism-related genes. It was reported that LEF1 is highly expressed in oligodendrocyte precursor cells and contributes to post-injury repair. Moreover, it regulates complement system genes (e.g., *C1S*) to influence the inflammatory response [141,142]. SP1, a ubiquitous transcription factor, regulates various injury-responsive genes such as RAB27A and activates promoters of cholesterol metabolism genes such as LCAT to participate in axonal repair [141]. During the chronic phase after SCI, transcriptome analyses of motor neurons have shown that transcription factors such as NF-κB and AP-1 family members regulate gene clusters associated with neuronal hyperexcitability, potentially contributing to the persistence of late-stage spasticity and plateau potential abnormalities [136,143].

Increasing evidence indicates that axonal regeneration can be significantly boosted by the deliberate manipulation of transcription factors via sophisticated regulation of neuroprotection, axonal growth, cytoskeletal remodeling, and neural circuit reconstruction. For example, Rho GTPases (RhoA, Rac, and Cdc42) act as molecular switches to control cytoskeletal dynamics. During regeneration, upregulation of microtubule-destabilizing proteins such as SCG10 and stathmin promotes axon elongation [123]. Moreover, co-expression of transcription factors, including Klf6/Nr5a2 and MEF2/Klf7, has been demonstrated to boost axonal sprouting in injured spinal cords, encourage the establishment of new neural pathways, and elevate the expression of regeneration-associated genes [144,145]. The combined expression of Ctcf with either Yy1 or E2f2 has also shown potential in stimulating axonal growth in vitro [138]. Importantly, overexpression of TFAM has been reported to significantly improve functional recovery, partly through regulating downstream targets such as IGF1, which support neuronal survival and outgrowth [146].

##### Role of Axon Guidance Molecules

Axon guidance molecules (AGMs), which encompass both permissive and inhibitory guidance molecules, are pivotal in orchestrating axonal growth and the reestablishment of neural circuits. The bidirectional character of AGMs suggests that their effects are contingent upon variables such as axon subtype, the injury microenvironment, and the availability of other signaling molecules [147]. Notably, Netrins stand out as quintessential axon guidance molecules. They interact with their receptors, DCC or UNC-5, to navigate axons towards their target regions, thereby fostering the formation of appropriate neural circuits [124]. In a parallel vein, Wnt family proteins have evidently participated in axonal regeneration post-SCI, thereby aiding in the reconstruction of neural networks and functional recovery [147]. On the flip side, repellent AGMs like Slits and Semaphorins see a marked upregulation in the injury environment. Although these molecules serve a vital role in preventing aberrant connectivity by directing axons away from non-target areas, their prolonged post-injury overexpression might ironically hamper regenerative axonal extension. This can lead to the creation of inhibitory barriers to neural repair [124,125]. Furthermore, the reactivation of Eph/ephrin signaling post-CNS injury plays a significant role in inhibiting axonal regeneration. It triggers growth cone collapse and manages astrocyte proliferation and glial scar formation, thus constraining neural repair via multiple mechanisms [148].

### 2.3. Summary of Neuroplasticity Mechanisms After SCI

**Figure 1:**
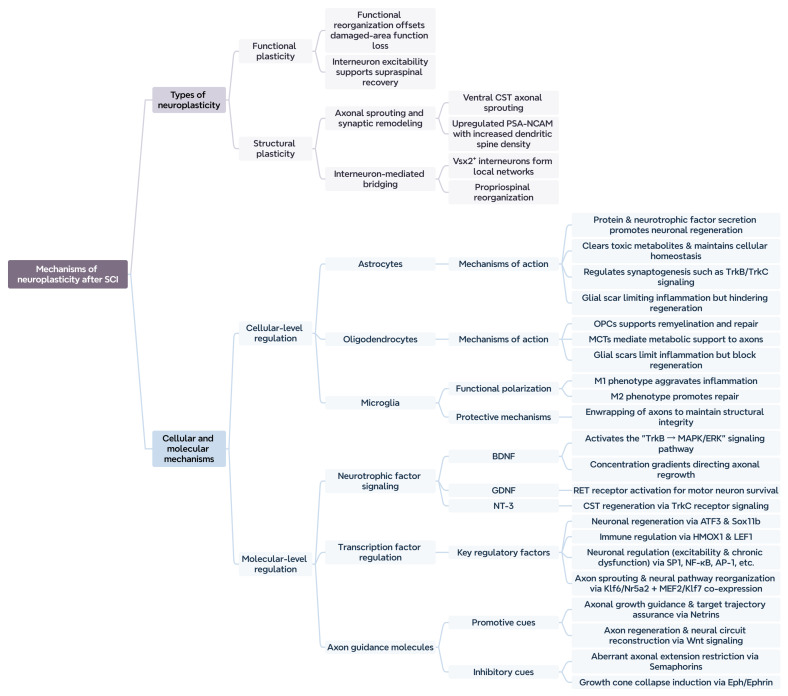
Mechanisms of neuroplasticity after SCI.

We conducted a preliminary evaluation of the potential mechanisms underlying neural plasticity following SCI (Figure 1). This will assist in the development and refinement of therapeutic strategies for SCI.

### 2.4. Other Critical Factors Affecting Neuroplasticity

The potential for neuroplasticity and recovery after SCI is influenced by patient age, the severity of injury, and the time after injury. The nervous system of younger patients is more plastic, with high levels of gene transcription, synaptic remodeling, and axonal elongation. Aging is associated with decreased metabolic activity of neurons, low levels of expression of regenerative signaling molecules, and reduced capacity for adaptation of the extracellular microenvironment, which reduces neuroplasticity [149,150]. It has been established that the human hippocampus maintains lifelong neurogenesis [151,152,153,154]. However, the ability of neurons in mammals to regenerate decreases with age [155,156]. Embryonic and immature mammalian neurons have a higher intrinsic growth capacity after injury than adult neurons. This is related to increased levels of intrinsic pro-regenerative factors (e.g., BDNF, NT-3) and decreased levels of inhibitory extrinsic factors (e.g., Nogo-A and MAG) [157]. Sarcopenia, inflammation, and other aging-related physiological changes also contribute to a decreased ability of nerve tissue to regenerate and adapt [158,159]. Immature neurons express pro-regenerative transcriptional profiles that are gradually turned off as they mature [160].

**Figure 2:**
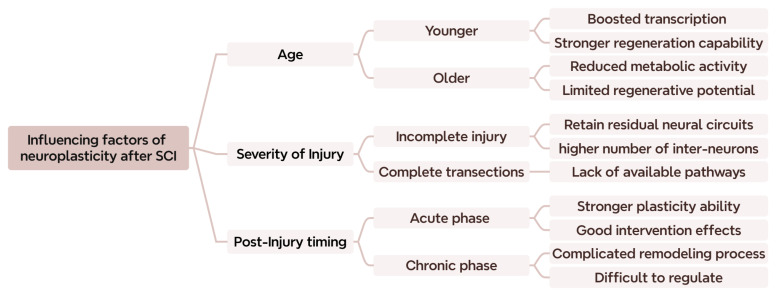
Influencing factors of neuroplasticity after SCI.

The severity of SCI significantly influences the degree of neuroplasticity and functional recovery, as it impacts the reorganization of neural circuits. Those with an incomplete SCI maintain some preserved neural pathways which can be enhanced or reorganized, thereby providing a larger substrate for plasticity and functional restoration [161]. Generally, the less severe the injury, the more likely the nervous system is to achieve functional reconstruction through adaptive mechanisms [158]. Research suggests that preserved interneurons and axons caudal to the lesion site promote compensatory plasticity and functional improvements, which are considerably limited in severe injuries [6,162]. Moreover, the structural and functional remodeling of corticospinal networks dynamically evolves with the completeness of the injury, profoundly influencing the overall potential for rehabilitation [10,11]. An overview of the factors influencing neuroplasticity after SCI is illustrated in Figure 2.

The period following SCI profoundly impacts neuroplasticity. In the acute phase, increased neuroplasticity is primarily attributed to acute-phase responses. Conversely, the chronic phase is characterized by intricate adaptive reorganization and synaptic recalibration [11]. Thus, initiating rehabilitation interventions at an early stage leverages the enhanced adaptive potential of the acute phase, leading to better functional recovery outcomes.

## 3. Strategies to Promote Neural Remodeling After SCI

The enhancement of neuroplastic alterations subsequent to SCI stands as a pivotal approach to ameliorating functional outcomes in affected individuals. The augmentation of neuroplasticity is fundamentally dependent upon four intrinsic biological mechanisms: the modulation of inherent neuronal signaling, the optimization of the extracellular microenvironment, the reconstruction of interrupted spinal pathways via neural stem cell transplantation, and the regulation of neuronal electrical activity [163]. In recent years, a wide range of therapeutic strategies, founded upon these mechanisms, has been meticulously developed with the express purpose of effectively activating and harnessing neuroplasticity to facilitate functional recovery.

### 3.1. Pharmacological Interventions

Pharmacological interventions following SCI not only alleviate complications but also show potential for improving neural repair and functional recovery [164]. Methylprednisolone (MP) is the sole FDA-approved corticosteroid for acute SCI. It mainly targets secondary injury cascades [165]. MP offers neuroprotection through various mechanisms. It suppresses the inflammatory response, diminishes free radical production, and inhibits lipid peroxidation [166,167]. These actions help limit secondary tissue damage after the initial insult. The National Acute Spinal Cord Injury Study (NASCIS), particularly the NASCIS II and III trials, substantiated that high-dose MP administration within 8 h of injury can moderate secondary injury mechanisms and slightly improve neurological outcomes [168]. Despite ongoing debates about its clinical effectiveness and potential adverse reactions, MP remains a pivotal therapeutic option for early intervention in acute SCI. It plays an important role in addressing secondary pathophysiological processes. Other agents, such as melatonin, estradiol, and atorvastatin, have shown promising neuroprotective and regenerative effects in preclinical research [164].

Melatonin, a hormone primarily secreted by the pineal gland, exhibits neuroprotective properties through its antioxidative and anti-inflammatory pathways [169]. Research has shown that mesenchymal stem cells (MSCs) preconditioned with melatonin enhance motor functional recovery in SCI *mice* by maintaining stable mRNA expression of ubiquitin specific peptidase 29 [170]. Additionally, the combined treatment of melatonin and treadmill training decreases neuroinflammation, mitigates neuronal deformation, augments neuronal differentiation, and bolsters post-SCI motor function [170].

Estrogen therapy has been shown to exhibit substantial neuroprotective effects in models of SCI [171]. Prior research has demonstrated that estradiol can enhance hindlimb motor function in ovariectomized *rats* through the inhibition of neuronal apoptosis and promotion of white matter preservation. This effect also extends to the reduction in apoptosis levels in both premenopausal and post-menopausal animal models [172]. Moreover, estrogen offers expansive neuroprotective benefits following SCI via various mechanisms. These include suppression of inflammatory pathways, inhibition of microglial activation, improvement of blood flow in damaged tissues, upregulation of anti-apoptotic proteins, and mitigation of post-traumatic calcium influx [173].

Atorvastatin, a widely prescribed drug for lowering cholesterol levels, has demonstrated neuroprotective potential in numerous instances [174]. Preclinical studies have indicated that atorvastatin can enhance neurological behavioral performance, improve biochemical indicators, and ameliorate histopathological status in *rabbit* models of ischemic SCI. These findings suggest the therapeutic potential of atorvastatin in the treatment of SCI [174].

In the pharmacological intervention for SCI, while several drugs exhibit certain neuroprotective or anti-inflammatory effects, their potential adverse reactions warrant caution. Methylprednisolone use in acute SCI has been linked to a heightened risk of serious complications, including infections, gastrointestinal bleeding, steroid-induced myopathy, hyperglycemia, and cardiovascular events [165,175,176]. Given these safety concerns and the inconsistent neural benefits, there is growing resistance to its routine clinical application. While estrogen therapy shows promise in neuroprotection for SCI, its application is constrained by safety considerations, primarily an elevated risk of thromboembolic events [177] and hormone-dependent cancers [178], especially with prolonged or high-dose exposure. Atorvastatin is generally well-tolerated in SCI patients, presenting rare instances of neuropathy and a myopathy rate of roughly 1–5% [179]. However, it may be associated with delayed motor recovery due to the inhibition of cholesterol synthesis [180].

### 3.2. Biomaterials

In recent years, the swift advancement of tissue engineering technologies has led to the amalgamation of biomaterials with medical science, offering novel approaches to the treatment of SCI [181]. Biomaterials in SCI therapy provide mechanical support, enable effective delivery of drugs and cells, and modulate the post-injury microenvironment. These effects together help improve tissue repair outcomes [182,183,184]. Specifically, biomaterials can fill lesion cavities, restore disrupted neural continuity, deliver stem cells or bioactive molecules, guide axonal regeneration, attract endogenous neural stem/progenitor cells, and inhibit the formation of glial scars [182].

Chitosan, a widely used natural biomaterial, shows excellent biocompatibility and bioactivity. Chitosan-based scaffolds promote tissue and vascular regeneration, reduce glial scar formation, and regulate inflammation. However, the fast degradation and excessive swelling associated with pure chitosan scaffolds necessitate their combination with other materials to enhance performance and prolong functional stability [181]. Research has revealed that implanting NT3-chitosan scaffolds into completely transected and removed thoracic spinal cord lesions in *rats* significantly activates endogenous neural stem cells (NSCs). This process stimulates their differentiation into mature neurons and facilitates the establishment of functional neural networks by reconnecting ascending and descending axonal tracts, leading to both sensory and motor functional recovery [185]. Further studies confirm that following lesion core clearance, NT3-chitosan scaffolds can foster axonal regrowth, reestablish severed connections of both ascending and descending tracts, and improve electrophysiological and motor outcomes. These findings suggest that NT3-chitosan may promote regeneration even in chronic SCI models [186]. A key challenge in chitosan-based scaffold design for SCI repair is achieving a degradation rate that matches the pace of neural regeneration. Premature degradation may lead to loss of structural support, while delayed degradation can trigger chronic inflammation or hinder tissue remodeling. Strategies such as chemical modification, polymer blending, and scaffold architecture optimization are being explored to precisely control degradation and enhance functional recovery.

With regard to synthetic biomaterials, propylene fumarate has garnered significant attention due to its superior mechanical strength and capacity to bolster tissue regeneration [182,187]. The tunable mechanical properties and effective drug delivery capabilities of hydrogels have made them a subject of extensive research. For instance, the integration of fibroblast growth factor 2, epidermal growth factor, and GDNF into polycaprolactone-based hydrogels not only provides structural support but also promotes adhesion, growth, and proliferation of neural cells [182]. Furthermore, three-dimensional alginate scaffolds containing neural stem cells and oligodendrocytes have demonstrated an enhancement in hindlimb motor function in models of SCI [188].

### 3.3. Gene Editing

Gene editing therapy represents a pioneering strategy for SCI repair, presenting an opportunity to address fundamental challenges in regenerative medicine. This technique seeks to meticulously modulate gene expression at the lesion site, fostering neural repair and functional restoration. A notable approach involves the viral introduction of developmental transcription factors, such as SOX2. These factors can convert reactive astrocytes into proliferating neuroblasts and stimulate their differentiation into mature neurons, facilitating the reestablishment of damaged neural networks [189]. Additionally, research has demonstrated that adeno-associated virus (AAV)-mediated overexpression of the presynaptic organizer fibroblast growth factor 22 enhances synapse formation in relay neurons. This process augments circuit plasticity and bolsters motor functional recovery, suggesting a novel avenue for early gene therapy intervention in synaptic repair post-SCI [190].

Leveraging these advancements, state-of-the-art gene editing technologies [191] have broadened the array of intervention methods for SCI treatment. These technologies target and modulate regeneration-inhibiting genes (e.g., phosphatase and tensin homolog deleted on chromosome ten, PTEN) and components of inflammatory pathways (e.g., TGF-β, TNF-α). Through this approach, not only are intrinsic neuronal regenerative programs activated, but glial scar formation is also suppressed, and the immune microenvironment is modulated. For example, a study employing AAV-rg-mediated retrograde transduction realized targeted PTEN deletion within spinal circuits, leading to a significant improvement in forelimb motor recovery in mice with cervical SCI. Nevertheless, sex differences and delayed pathological effects were noted, indicating a potential trade-off between regenerative promotion and adverse outcomes in PTEN modulation [192]. Moreover, the TGF-β signaling pathway, central to glial scar formation, can be effectively targeted to reduce aberrant activation of glial cells and the production of CSPGs, thus mitigating their inhibitory impact on axonal regeneration [193]. Subsequent research has demonstrated that the gene deletion of the pro-inflammatory cytokine TNF-α markedly diminishes neuronal death and tissue damage in the lesion area, ultimately enhancing functional recovery outcomes [194,195].

Gene therapy presents significant promise for injury repair; however, its practical application is confronted with numerous challenges. These encompass the complexity of SCI pathology, which necessitates multi-targeted interventions [189,196], the restricted intrinsic regenerative capabilities of adult CNS neurons [196], and the existence of extrinsic inhibitory factors such as myelin-associated inhibitors and CSPGs [197]. Moreover, issues related to the safety and efficiency of gene delivery, the risk of off-target effects, and immune responses [198], and the absence of consensus on optimal gene targets [199] further impede clinical translation. Although encouraging results have been achieved in preclinical studies, translating these findings into effective human therapies remains hindered by biological differences and the complexity of human SCI. Achieving meaningful functional recovery will likely require combining gene therapy with other therapeutic strategies.

### 3.4. Stem Cell Therapy

Stem cell-based therapies hold considerable therapeutic promise for SCI, as they can foster neural repair, modulate the local injury microenvironment, and reconstruct damaged neural circuits, thereby substantially enhancing neuroplasticity [200,201]. NSCs and induced pluripotent stem cells (iPSCs) have shown the capability to differentiate into neurons, astrocytes, and oligodendrocytes. These cells effectively replace damaged cells, contributing to remyelination and axonal regeneration [202,203,204,205]. Beyond direct cell replacement, stem cells secrete neurotrophic factors such as BDNF and GDNF, which support neuronal survival, axon outgrowth, and synaptic plasticity [122,206,207]. Both NSCs and MSCs can influence the injury microenvironment by reducing inflammation, minimizing glial scar formation, and degrading inhibitory components like CSPGs. This creates a more conducive environment for axonal extension [208]. Moreover, transplanted stem cells can activate endogenous neural networks, encourage collateral sprouting and synaptic reorganization of residual neurons, and further boost activity-dependent neural plasticity when combined with rehabilitation training [203,209,210]. Clinically, NSC transplantation has led to improvements in motor and sensory functions in chronic SCI patients. The Mayo Clinic’s CELLTOP clinical trial reported that treatment with adipose-derived MSCs resulted in at least one-grade improvement on the American Spinal Injury Association Impairment Scale in 70% of patients, accompanied by reductions in inflammation and neuropathic pain [211]. Animal studies have demonstrated that implanting NSCs within polymer scaffolds can promote regeneration of the CST and long-term functional recovery (up to one year) in *rats* [205]. Additionally, iPSC-derived NSCs have successfully differentiated into neurons in *rodent* SCI models, resulting in a significant improvement in locomotor function [212].

Stem cell therapy faces numerous challenges, including the selection of optimal cell types [213], overcoming the hostile injury microenvironment [214], and managing risks such as tumorigenesis, immune rejection, and variable efficacy [200]. Ethical concerns, particularly those surrounding embryonic stem cells [200], as well as unresolved issues related to delivery methods and timing [196,215], further complicating its clinical translation.

### 3.5. Rehabilitation and Physical Therapy

Rehabilitation and physical therapy stand as pivotal interventions for fostering neurological recovery post-SCI. These methodologies bolster neuroplasticity, augment [216], and expedite the recovery of motor functions [217], thereby elevating the overall quality of life for patients. Empirical evidence underscores that moderate-to-high-intensity exercise, conducted 2–3 times weekly, markedly improves physical fitness and muscle strength in individuals with chronic SCI. Furthermore, it serves as a potent countermeasure against disuse-induced muscle atrophy [216,217]. Prompt rehabilitation is imperative not only for averting joint contractures but also for preserving bone mineral density and upholding the efficiency of the respiratory and gastrointestinal systems [218]. In the contemporary therapeutic landscape, advancements in rehabilitation technology have broadened the horizon of treatment possibilities. Emerging techniques such as transcranial magnetic stimulation (TMS), functional electrical stimulation (FES), and robot-assisted treadmill training are demonstrating enhanced efficacy in rehabilitation protocols [219].

#### 3.5.1. Treadmill Training

Treadmill training is a well-established intervention known to promote neural plasticity through various mechanisms. It aids in the remodeling of spinal circuits and the regeneration of axons, thereby enhancing functional recovery. The regulatory mechanisms involved include (1) an upregulation of GAP-43, which stimulates axonal sprouting and synaptogenesis while maintaining synaptic function [220]; (2) the activation of BDNF expression in lumbar motor neurons, promoting both neuronal survival and synaptic reorganization [221,222]; (3) a structural remodeling of motor neuron dendrites, leading to an increase in dendritic length and synaptic density, thus improving synaptic connectivity [221]; (4) a remodeling of the CST, resulting in an increased synaptic density and enhanced function [220].

Bodyweight-supported treadmill training utilizes a harness or suspension device to support a portion of a patient’s body weight, allowing them to walk on the treadmill without bearing their full body weight [223,224,225,226]. When integrated with robotic assistance, these treadmill systems can provide precise, individualized control of gait kinematics. They are particularly effective for enhancing walking speed, distance, strength, range of motion, and activity levels in patients with incomplete SCI [227,228,229].

Although effective, the results of treadmill training are dependent on the injury severity, timing of training, and inflammatory state. The effects of treadmill training are limited in patients with complete SCI [222]. Furthermore, the initiation of treadmill training at acute stages of inflammation might be detrimental to its effects. Indeed, in *mice*, early treadmill training during a period of heightened inflammation failed to produce long-term improvements, whereas delaying the initiation of treadmill training until after the inflammation resolved resulted in substantial hindlimb recovery [230]. Also, the neuroplasticity induced through treadmill training is partially reversible, as previously regained locomotor function was lost in spinalized *cats* after 12 weeks without training [231], highlighting the importance of sustained rehabilitation.

#### 3.5.2. Photobiomodulation

Photobiomodulation (PBM) represents a promising non-invasive therapeutic approach. Recent studies have underscored its multi-target and multi-mechanism capabilities in facilitating functional recovery following SCI. PBM synergistically operates through several mechanisms, including neuroprotection, anti-inflammatory responses, regulation of mitochondrial function, and pain alleviation. These combined effects notably enhance neural plasticity and support functional reconstruction after injury [219,232,233,234,235].

PBM suppresses neurotoxic A1 astrocytes while activating the neuroprotective A2 phenotypes. It facilitates local microenvironmental repair by upregulating neurotrophic factors such as basic fibroblast growth factor and transforming growth factor β (TGF-β), thereby promoting axonal regeneration and neural circuit reconstruction [235]. PBM also activates critical signaling pathways, including Notch3, Slit1/Robo2, and Sema3g, to enhance axonal growth and connectivity [234]. With respect to cellular energy metabolism and mitochondrial function, PBM can downregulate pathways associated with apoptosis and metabolic stress (such as oxidative phosphorylation), consequently reducing neuronal death and improving mitochondrial function. By activating the AMPK/PGC-1α/TFAM pathway, PBM can promote the recovery of mitochondrial bioenergetics and support the high energy metabolism requirements during neural repair [234,236]. Furthermore, PBM modulates calcium signaling and the cAMP pathway to improve axon extension and synaptic connection formation, and regulates microglia/macrophage activation to suppress excessive inflammation [234,237]. PBM has been demonstrated to reduce lesion volume, increase neuronal survival, and enhance motor recovery [235,238], with these effects closely associated with increased neuroplasticity.

#### 3.5.3. Electrical Stimulation

Electrical Stimulation (ES) is a prevalent clinical rehabilitation method that augments proprioceptive feedback by activating large-diameter afferent fibers in the spinal cord. This process encourages the formation of functional neural circuits and instigates plastic changes to enhance motor control [239]. The primary forms of ES include peripheral nerve stimulation, FES, and epidural electrical stimulation (EES). Peripheral nerve stimulation targets peripheral nerves to alleviate pain and improve motor function [239]. FES delivers low-level electrical pulses via surface electrodes to induce muscle contractions. It is widely used to enhance limb movement, alleviate foot drop, increase strength, and restore autonomic functions such as bladder control [240]. EES involves implanting electrodes into the epidural space to directly activate spinal circuits and central pattern generators in the lumbosacral cord, enabling weight-bearing stepping [241]. Emerging evidence suggests that targeted, non-invasive EES can significantly improve motor outcomes in individuals with varying degrees of paralysis [242]. Greiner et al. [243] employed computational modeling and animal experiments to show that cervical EES can achieve segmentally selective recruitment of upper-limb motoneurons through the activation of sensory afferent fibers, with muscle responses dynamically modulated during voluntary movement. Our laboratory’s recent findings indicate that combining ES with PBM enhances the specificity and effectiveness of motor recovery by increasing activation of target muscle groups after SCI [244,245].

#### 3.5.4. Transcranial Stimulation

Transcranial stimulation represents a promising non-invasive neuromodulatory strategy for SCI rehabilitation. This technique has the potential to enhance neuroplasticity, facilitate motor recovery, and mitigate complications such as spasticity and neuropathic pain. The most frequently employed modalities are transcranial direct current stimulation (tDCS) and TMS.

tDCS has been shown to influence cortical excitability and spinal network activity. When applied over the motor cortex after SCI, tDCS can re-establish corticospinal neuron synchrony and alter microglial morphology [246,247]. Cho et al. [248] demonstrated that glutamatergic neurons in the lateral hypothalamus play a significant role in the recovery of gait performance after an incomplete SCI. Consequently, they developed a deep brain stimulation-based intervention that enhanced locomotor ability by remodeling descending projections.

TMS is a technology that influences neuronal activity of a target site and its associated network by inducing local magnetic field alterations. This elicits plastic changes in specific brain regions [249]. TMS not only offers therapeutic potential for motor function recovery and pain relief, but can also be paired with motor evoked potential technology to assess the functional state of the corticospinal tract. This enables an evaluation of the integrity and recovery potential of the neural pathways in SCI patients [249,250]. Repetitive TMS (rTMS) has demonstrated beneficial effects in patients with incomplete SCI. When administered over the primary motor cortex or dorsolateral prefrontal cortex, rTMS significantly improves hindlimb strength, gait, posture, and neuropathic pain [251,252,253].

#### 3.5.5. Sensory Stimulation

Tactile and proprioceptive feedback are integral to motor control. Research has demonstrated the critical role of sensory input in reestablishing voluntary control over affected muscles and fostering functional recovery post-SCI [11,254,255]. Sensory stimulation therapy (SST) represents a burgeoning field in SCI rehabilitation, emphasizing the restoration of sensory function and the enhancement of sensorimotor integration. Spinal dorsal stimulation has exhibited potential in rodent and primate models by eliciting artificial sensations, thereby laying a foundation for neural prostheses to revive sensory perception [256]. While transcutaneous spinal cord stimulation is predominantly employed for motor rehabilitation, it also indirectly bolsters sensory function via dorsal root and motor circuit activation, leading to improved upper limb functionality in patients with chronic incomplete SCI [254]. Multisensory integration, such as the utilization of auditory stimuli to offset sensory deficits, highlights the promise of cross-modal rehabilitation techniques [257]. Nevertheless, SST continues to grapple with substantial challenges such as the absence of extensive clinical validation and a limited comprehension of its underlying mechanisms.

#### 3.5.6. Summary of Rehabilitation and Physical Therapy After SCI

While rehabilitation and neuromodulation therapies offer potential benefits for SCI recovery, their application is constrained by considerable practical, physiological, and technical challenges. These impediments encompass extensive resource requirements, exemplified by treadmill training, the limited clinical evidence backing PBM [234], the muscle fatigue and technical intricacy linked to ES [258], the inconsistent and transient impacts of transcranial stimulation [249,253,259], and the individual variability in responses to sensory stimulation [257]. The refinement of protocols and the formulation of personalized multimodal strategies will be imperative to enhance therapeutic results.

### 3.6. Comparative Analysis of Therapeutic Strategies Targeting Neuroplasticity

The exponential progression of regenerative medicine, molecular biology, and rehabilitation science has led to a diverse array of therapeutic strategies for SCI. These now encompass pharmacological interventions, biomaterials, gene editing, stem cell therapy, and physical rehabilitation. Each approach varyingly targets and regulates neuroplasticity-related mechanisms, showcasing individual advantages and limitations (Table 1). This reflects the complexity and multidimensional requirements of SCI treatment at the molecular, cellular, and systemic levels.

Pharmacological therapy is paramount during the acute phase of SCI, chiefly facilitating inflammation modulation and neuroprotection. Agents such as corticosteroids can diminish inflammatory responses, mitigate secondary tissue damage, and alleviate symptoms like pain, spasticity, and mood disorders. Although these treatments are readily available, they often fall short in reversing neuronal damage and exhibit limited long-term efficacy [164]. Biomaterial-based interventions offer structural scaffolds to support axonal regeneration, span lesion voids, and allow targeted delivery of therapeutic agents or cells. Their biocompatibility and biodegradability render them potential tools for enhancing structural neuroplasticity, encompassing axonal reconnection and synaptogenesis. Nevertheless, concerns persist regarding inflammatory reactions, cytotoxic degradation by-products, and persistent challenges in material design and functional integration [183,184]. Gene editing technologies allow for the precise manipulation of neuroplasticity-associated genes by activating innate regenerative pathways and altering extracellular inhibitory signals. These methods hold considerable promise for sustained molecular-level neuroprotection and regeneration. However, gene therapy is predominantly preclinical, limited by challenges such as suboptimal delivery efficiency, unintended off-target effects, and an underdeveloped clinical translation [199,260]. Stem cell therapies have garnered considerable interest due to their regenerative capabilities and immunomodulatory attributes. When transplanted, stem cells can differentiate into neurons and glial cells, modulate the post-injury microenvironment, and enhance neuroplasticity. However, ethical considerations, potential tumorigenicity, and variability in therapeutic outcomes necessitate further optimization of stem cell sources and clinical approaches [260]. Rehabilitation training and physical therapy stand as core interventions for boosting activity-dependent neuroplasticity in individuals with SCI. These techniques aid in synaptic remodeling, CST reorganization, and activation of residual sensorimotor circuits, leading to improved motor function, relief from chronic pain, and psychosocial support. While their safety and general applicability are commendable, they present limitations such as modest regenerative impact, substantial time and resource requirements, and pronounced inter-individual variability in outcomes.

## 4. Conclusions and Future Perspectives

SCI is a severe neurological condition frequently leading to substantial motor and sensory deficits. This review offers a systematic overview of the mechanisms and manifestations of neuroplasticity post-SCI, delves into the pivotal factors affecting neural remodeling, and evaluates the effectiveness of an array of therapeutic interventions ranging from pharmacological treatments and biomaterials to gene editing, stem cell therapies, and physical rehabilitation—in enhancing neuroplasticity. While each method shows distinct therapeutic advantages, the impact of any singular intervention remains constrained. Future investigations should prioritize a deeper understanding of the intrinsic processes driving neuroplasticity post-SCI and delineating the unique benefits of each therapeutic approach. Additionally, research emphasis should be directed towards devising multimodal and synergistic treatment paradigms and conducting expansive clinical trials. Through the amalgamation of tailored rehabilitation programs with cutting-edge therapeutic methods, we can potentially optimize the capabilities of neuroplasticity, markedly enhancing functional recovery and the quality of life for those affected by SCI.

## Figures and Tables

**Table 1:** Characteristics and outcomes of various SCI treatment approaches.

Type of Intervention	Effects on Neuroplasticity	Advantages	Limitations
Pharmacological Interventions	Indirectly activate neuroplasticity through anti-inflammatory and neuron protection	(i) The operation is relatively simple and has a wide range of applications (ii) Suppress inflammation, reduce tissue damage, and alleviate complications such as pain and spasms	(i) The side effects are significant (ii) Difficult to reverse neural damage
Biomaterials	Provide dual support of physical scaffolding and biochemical signaling to directly promote structural plasticity and neural guidance	(i) Good biocompatibility and degradability (ii) Promotes cell adhesion and axon regeneration (iii) Serve as a carrier for drugs or cells to achieve targeted therapy	(i) Induce inflammatory reactions or degradation toxicity (ii) Challenges in material design
Gene Editing	Activate the mechanisms of axonal regeneration and synapse formation at the molecular level	(i) Precisely regulating key genes that promote neuroprotection and regeneration at the molecular level (ii) Potential for long-term therapeutic application	(i) Still in the pre-clinical stage (ii) Low delivery efficiency, off-target effects, and immature technology
Stem Cell Therapy	Achieve the reconstruction of structural and functional plasticity by implanting cells to construct conduction pathways	(i) Differentiating into neurons / glial cells (ii) Improving the microenvironment (iii) promoting regeneration and regulating immunity	(i) Ethical controversies and tumor risks (ii) Therapeutic effect is unstable (iii) Need to optimize the strategy for the source and use of stem cells
Rehabilitation and Physical Therapy	Activate activity-dependent pathways, induce synaptic remodeling, and neural network function reconstruction	(i) Significantly improve motor function and quality of life (ii) Alleviate chronic pain	(i) The ability to repair is limited (ii) The treatment is contingent upon time and resources (iii) Significant variations among individuals
